# Bacteriophage-Induced Lipopolysaccharide Mutations in *Escherichia coli* Lead to Hypersensitivity to Food Grade Surfactant Sodium Dodecyl Sulfate

**DOI:** 10.3390/antibiotics9090552

**Published:** 2020-08-28

**Authors:** Zeyan Zhong, Jean-Guillaume Emond-Rheault, Sudhakar Bhandare, Roger Lévesque, Lawrence Goodridge

**Affiliations:** 1 Food Science and Agricultural Chemistry, McGill University, Sainte-Anne-de-Bellevue, QC H3X 3V9 Canada; zeyan.zhong@mail.mcgill.ca; 2 Institut de Biologie Intégrative et des Systèmes (IBIS), Université Laval, Quebec City, QC G1V 0A6, Canada; jean-guillaume.emond-rheault.1@ulaval.ca (J.-G.E.-R.); rclevesq@ibis.ulaval.ca (R.L.); 3 Operations Branch, Canadian Food Inspection Agency (CFIA), Charlottetown, PE C1E 1E3, Canada; vetsudhakar@bristolalumni.org.uk; 4 Food Science Department, University of Guelph, Guelph, ON N1G 2W1, Canada

**Keywords:** lytic bacteriophage, *Escherichia coli*, lipopolysaccharide, combined therapy, food safety

## Abstract

Bacteriophages (phages) are considered as one of the most promising antibiotic alternatives in combatting bacterial infectious diseases. However, one concern of employing phage application is the emergence of bacteriophage-insensitive mutants (BIMs). Here, we isolated six BIMs from *E. coli* B in the presence of phage T4 and characterized them using genomic and phenotypic methods. Of all six BIMs, a six-amino acid deletion in glucosyltransferase WaaG likely conferred phage resistance by deactivating the addition of T4 receptor glucose to the lipopolysaccharide (LPS). This finding was further supported by the impaired phage adsorption to BIMs and glycosyl composition analysis which quantitatively confirmed the absence of glucose in the LPS of BIMs. Since LPSs actively maintain outer membrane (OM) permeability, phage-induced truncations of LPSs destabilized the OM and sensitized BIMs to various substrates, especially to the food-grade surfactant sodium dodecyl sulfate (SDS). This hypersensitivity to SDS was exploited to design a T4–SDS combination which successfully prevented the generation of BIMs and eliminated the inoculated bacteria. Collectively, phage-driven modifications of LPSs immunized BIMs from T4 predation but increased their susceptibilities as a fitness cost. The findings of this study suggest a novel strategy to enhance the effectiveness of phage-based food safety interventions.

## 1. Introduction

Antimicrobial resistance (AMR) is now recognized as a global health crisis that demands immediate intervention. A high-profile report published by British economist Jim O’Neil stressed that AMR would be more life-threatening than cancerous diseases by 2050, causing 10 million deaths a year globally. Almost 90% of this fatality is expected to come from developing countries in Asia and Africa [1]. A panel of experts from the Council of Canadian Academies also forecasts that the resistance rates to first-line antimicrobials would likely rise from 26% in 2018 to 40% by 2050, leading to a cumulative loss of 396,000 lives in Canada [2]. To slow down the wheel, the World Health Organization (WHO) has implemented a global action plan on AMR, aiming to improve public awareness, strengthen knowledge through surveillance and research, reduce the incidence of infection, optimize the use of antimicrobials and increase investment in new alternative medicine development [3].

Based on the gathered data from AMR surveillance systems like GLASS [4], WHO identified seven bacteria that confer high resistance rates to drugs commonly used for bacterial infectious diseases; among these species, the AMR situation of *Escherichia coli* (*E. coli*) is considered as the most critical [5]. Originally, this bacterium was merely a benign member of the commensal community in human and animal gastrointestinal tracts and intrinsically susceptible to many antimicrobial agents [6]; however, due to the increasing demand of animal protein, misuse and abuse of antibiotics in agricultural practices facilitate the acquisition of antibiotic genes in bacterial communities through horizontal gene transfer, consequently leading to the emergence of multiantibiotic-resistant *E. coli* strains [7]. It is estimated that infection caused by various multidrug-resistant *E. coli* strains will lead to more than 3 million deaths by 2050 [1]. Recently, Poirel et al. summarized the AMR found in *E. coli* isolates from human and animal sources to different antibiotic classes [6]. Of note, this widespread AMR is undermining the effectiveness of critically important antimicrobials used in common infection treatments. For example, while cephalosporins and fluoroquinolones are considered as the first-line agents to treat lower urine tract infection (UTI) caused by the uropathogenic *E. coli* [8,9], resistance to these compounds has been reported repeatedly [7,10,11,12] and predicted as a continuous growing trend in the next decade [13,14]. Since the high AMR prevalence is accelerating the extinction of effective antibiotics and limiting options for treatments, novel solutions are in great demand.

Phages, the viruses that can infect and kill bacteria, are considered as a promising alternative for antibiotics [15,16]. These microorganisms have gained popularity because of their unique characteristics as an antimicrobial agent. Approximately 10^31^ phage particles distribute ubiquitously in aquatic environments, gastrointestinal tracts in animals and other natural habitats on Earth [17], offering an immense reservoir for screening the proper bactericidal agents. Unlike the static effect of antibiotics, phages are continuously evolving with their bacterial hosts in an antagonistic manner. In other words, the dynamic interaction between phages and hosts allows the constant generation of new variants that can overcome resistance and infect bacteria. All these features advocate phages as a valuable candidate for developing antibacterial agents. In fact, a number of phage-based antimicrobial products now have been incorporated in food processing as a safety intervention, targeting foodborne pathogens such as *Listeria monocytogenes*, *Salmonella* and *E. coli* O157:H7 [18,19].

Remarkably, phage resistance has also been linked to the restoration of bacterial sensitivity to antimicrobial agents such as antibiotics. Chan et al. evaluated the antibiotic susceptibilities of *Pseudomonas aeruginosa* mutants that are resistant to lytic phage OMKO1, which absorbs to the outer membrane protein M (OprM) of the multidrug efflux systems’ MEX as a receptor site [20]. The results showed that these OMKO1-insensitive mutants were more susceptible to ciprofloxacin, tetracycline, ceftazidime and erythromycin than the parental strains, possibly due to the phage resistance-associated alterations in receptor OprM [20]. This trade-off between phage resistance and increased antibiotic sensitivity was also further explored using phage–antibiotic synergy to control uropathogenic *E. coli* isolates. The study conducted by Valério et al. [21] demonstrated that while using either phage ECA2 or ciprofloxacin (0.05 mg/L) alone showed a limited suppression effect on cell growth after 8 h incubation in urine samples, the combination of phage ECA2 and same amount of ciprofloxacin was able to eliminate the bacteria after 4 h incubation in the same environment. However, in an attempt of using phages to control *E. coli* in a food matrix, further studies are needed to characterize the genetic and phenotypic changes of these bacteriophage-insensitive mutants (BIMs) and evaluate whether the combination of phage and other food-grade agents can suppress phage resistance and confer a synergic antimicrobial effect.

**Figure 1:**
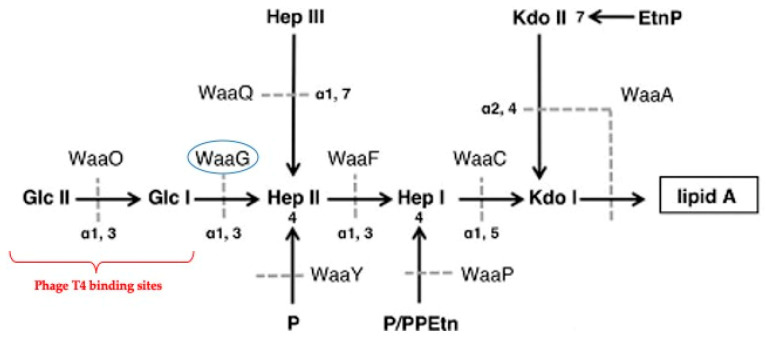
Structures of LPS in the wildtype *E. coli* B strain. Horizontal and vertical arrows indicate the main backbone and branches, respectively. Dotted lines represent the reactions catalyzed by glycosyltransferases in waa operon. The abbreviations are as follows: Glc, glucose; Hep, L-glycero-D-manno-heptose; Kdo, 3-deoxy-D-manno-oct-2-ulosonic acid; P, phosphate; P/PPEtn, phosphate or 2- aminoethyl diphosphate; and EtnP, ethanolamine phosphate. The specific receptor sites of phage T4 and glucosyltransferase waaG are highlighted in the red bracket and blue circle, respectively. This figure was adapted from the publication of Washizaki et al. [23].

In this work, by using the classic model of *E. coli* B and phage T4, we genomically and phenotypically characterized the BIMs isolated from *E. coli* B in the presence of phage T4 and evaluated the antibacterial efficacy of the combination of phage T4 and the food-grade surfactant sodium dodecyl sulfate (SDS). Given that previous studies had shown that phage T4 recognizes and binds to the exposed glucose terminus in the outer core of *E. coli* B’s LPS (Figure 1) as the only receptor site [22,23], six isolated BIMs (ZZa0, ZZa1, ZZa2, ZZa3, ZZa4 and ZZa5) were whole-genome sequenced and examined for their structural modifications in the receptor LPS. Loss of terminal glucose residues in this LPS in the presence of T4 was found to confer phage resistance and also sensitize bacteria to different substrates, especially SDS, suggesting a novel approach of combining phages with a food-grade surfactant to prevent the emergence of phage resistance, one of the major challenges of phage application.

## 2. Results

### 2.1. Phage T4 Resistant Mutant Isolation and Phage Adsorption Assays

**Figure 2:**
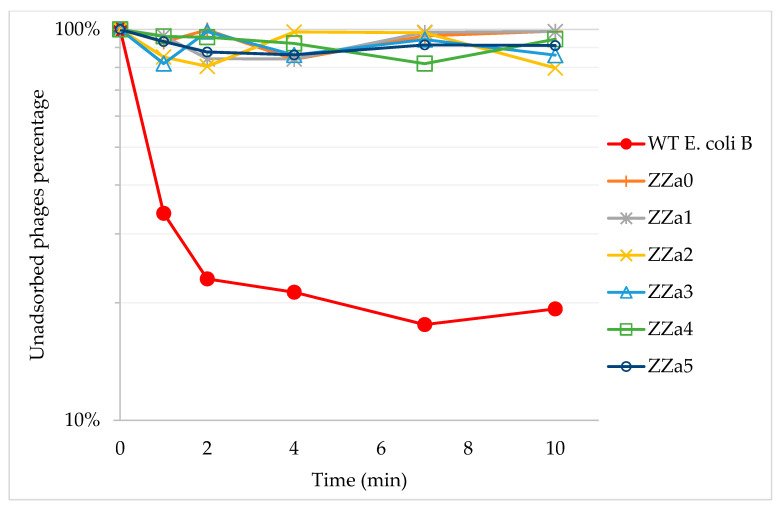
Adsorption kinetics of phage T4 to wildtype *E. coli* B and isolated BIMs, shown as unadsorbed phage percentages. Each data point was generated using the average results from three measurements.

Wildtype (WT) *E. coli* B ATCC 11303 was challenged by phage T4 at different titre levels. Six BIMs, i.e., ZZa0 (accession no. PQWJ00000000), ZZa1 (accession no. PQWI00000000), ZZa2 (accession no. PQWH00000000), ZZa3 (accession no. PQWG00000000), ZZa4 (accession no. PQWF00000000) and ZZa5 (accession no. PQWE00000000) were derived from infection by 10^9^, 10^8^, 10^7^, 10^6^, 10^5^ and 10^0^ PFU of phage T4, respectively. Phage resistance were further evidenced by phage adsorption assays (Figure 2). While adsorption to WT *E. coli* B occurred immediately and only 20% of phages left at the end, the titre of the free phage remained similar to the initial level when T4 was proliferated with six BIMs. Based on formula (1), the calculated adsorption rate constant k of WT *E. coli* B was 1.64 × 10^−9^ mL/min, while T4 adsorbed to BIM ZZa0, ZZa1, ZZa2, ZZa3, ZZa4 and ZZa5 at a rate of 1.09 × 10^−11^, 1.25 × 10^−11^, 2.26 × 10^−10^, 1.51 × 10^−10^, 5.79 × 10^−11^ and 9.51 × 10^−11^ mL/min, respectively, suggesting that these BIMs would be at least 7.26 times less likely than the wildtype to be infected by phage T4. Collectively, these findings suggest that phage T4 absorbed promptly to the WT strain but might not be able to attach to the BIMs.

### 2.2. Identification of Genetic Mutations Associated with phage Resistance

**Figure 3:**
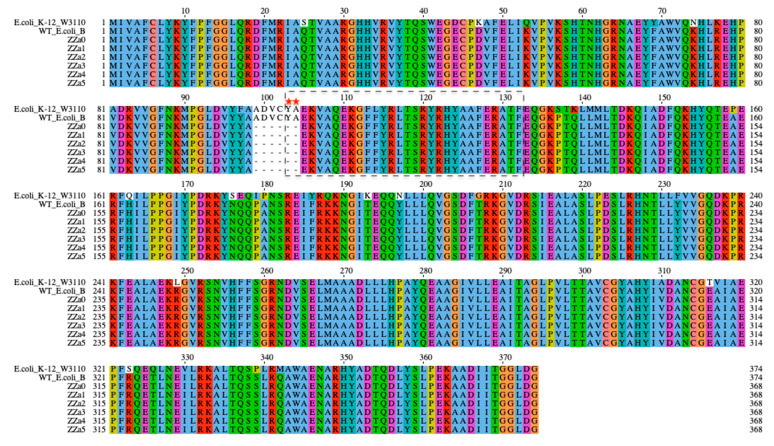
Amino acid sequences of WaaG of *E. coli* K-12 W3110 (PDB accession number 2IW1), the wildtype *E. coli* B and six bacteriophage-insensitive mutants (BIMs). Sequences were aligned using Clustal Omega and visualized by Jalview. The black box highlights the putative membrane-interacting region (MIR). The red asterisks signify the two amino acids deleted in the putative MIR of BIMs’ waaG. Amino acids were colored by the Clustal X scheme (orange: G; yellow: P; pink: C; red: K and R; magenta: E and D; cyan: H and Y; green: S, T, N and Q; blue: A, I, L, M, F, W and V). Gap is indicated by a dash.

Mapping to the sequence of wildtype *E. coli* B, single nucleotide polymorphism (SNP) analysis identified a mutated gene, *waag*, in operon waa of all BIMs consistently. Gene *waaG* is involved in the biosynthesis of LPSs, encoding an alpha-1,3-glucosyltransferase WaaG which catalyzes the transfer of a single glucose residue from an activated uridine diphosphate-D-glucose (UDP-Glc) to the nonreducing end of the inner core of the LPS (see Figure 1) [24,25]. A total of 374 and 368 amino acid residues were identified in the WaaG sequence of wildtype and BIMs, respectively (Figure 3). The six-amino acid (ADVCYA) difference was due to a deletion located at residues 99 to 104 found in the WaaG sequence of all BIMs. As a result, the mutated glucosyltransferase WaaG might be inactivated, subsequently disabling the addition of glucose, the phage receptor site, to the core area of the LPS.

### 2.3. LPS sugar Composition Analysis

Comparative gene alignments have shown that the gene encoding glucosyltransferase WaaG was mutated in all the BIMs, and the sugar composition of LPSs extracted from these mutants might have a different profile from the wildtype. Therefore, LPS sugar composition analysis was conducted using gas chromatograph mass spectrometry (GC-MS) to quantitatively compare the glycosyl profiles of the wildtype *E. coli* B and one of the BIMs, ZZa3, delineating the impact of WaaG mutation on LPS integrity.

**Figure 4:**
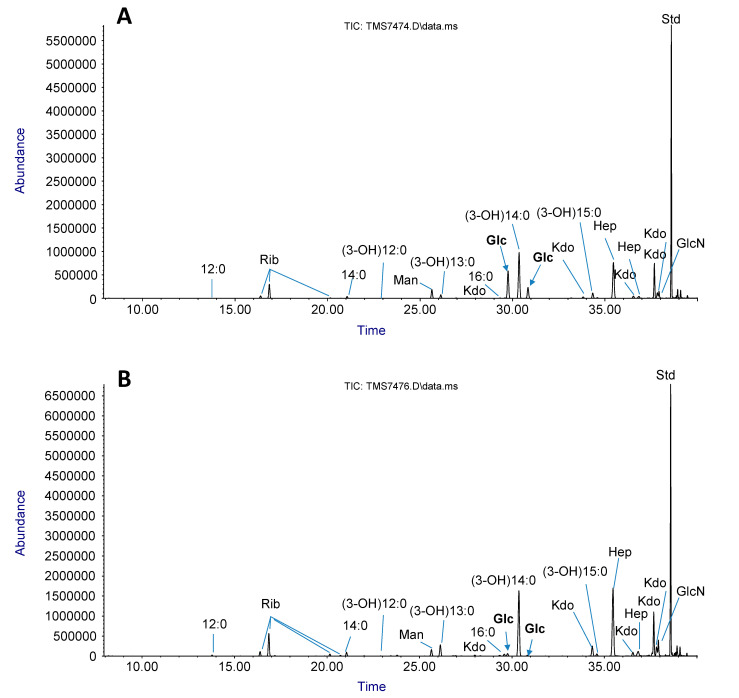
Sugar and fatty acid composition chromatograms of LPSs isolated from WT *E. coli* B (**A**) and BIM ZZa3 (**B**). A significant drop in the content of Glc in the ZZa3 mutant (Panel B) in comparison with the parental strain (Panel A) is highlighted (bold font and arrows).

Approximately 2 g of cells was harvested from 1 L of overnight culture and used to extract LPSs. After the purification process, 400 µg dry weight of LPS was collected to perform comparative chemical analysis by GC-MS. The glycosyl composition profiles of WT and ZZa3 are shown in Figure 4 and quantified in Table 1. As shown in Figure 1, the main sugar moieties present in both the wildtype and ZZa3 LPS were Glc, Hep, Kdo and GlcN (Figure 4). The presence of Rib, however, might be due to the residual ribose nucleic acid that was not fully removed during ultracentrifugation. A striking difference was observed in the Glc content where the relative mole percentage was 17.8% in the WT LPS, while only 1% was detected in the ZZa3 LPS (Table 1). Due to the significant drop in glucose content, the relative percentages of heptose and glucosamine were increased proportionally. Together, these findings implied that BIM ZZa3 was likely producing a truncated LPS where the glucose residues are absent from the core area due to the gene *waaG* mutation triggered by phage T4 infection. In addition to sugar residues, the fatty acid profiles were also shown in the chromatograms. Based on the relative peak area, the LPS of both samples consisted of mainly hydroxylated fatty acid chain (3-OH)14:0 and small amounts of (3-OH)15:0, (3-OH)12:0 and (3-OH)13:0, 12:0, 14:0 and 16:0, demonstrating acylation heterogeneity in lipid A. However, the mole percentage of each fatty acid was not measured in this study.

### 2.4. Minimum Inhibitory Concentration (MIC) Assay

It is noteworthy that LPSs play a crucial role in maintaining the integrity of the OM structure and phage-induced mutations in WaaG subsequently resulted in LPS truncation and membrane destabilization, as a cost of phage resistance. Therefore, the structural integrity of the WT and BIMs were evaluated by measuring the cross-membrane permeability of different substrates, including ampicillin (penicillin), kanamycin (aminoglycoside), novobiocin (aminocoumarin), polymyxin B (polypeptide), EDTA (membrane permeabilizer), sodium cholate (bile salt) and SDS (food-grade surfactant) (Table 2). Overall, mutants showed higher sensitivities to most of the substrates than the WT strain. An exception was made for the tolerance to novobiocin which was not affected by the *waaG* mutation. For ampicillin, kanamycin, polymyxin B and EDTA, BIMs showed at least 50% lower MIC values. Strikingly, the WT was relatively impermeable to SDS (MIC > 200 mg/mL), but the phage-resistant mutants conferred hypersensitivity (MIC ≤ 3 mg/mL) to this food-grade surfactant. The overall increased sensitivity to the tested compound demonstrated that the OM structure of BIMs was destabilized due to the phage-induced LPS truncation which permeabilized the cell to the surrounding environment.

### 2.5. Phage Resistance Inhibition Test

**Figure 5:**
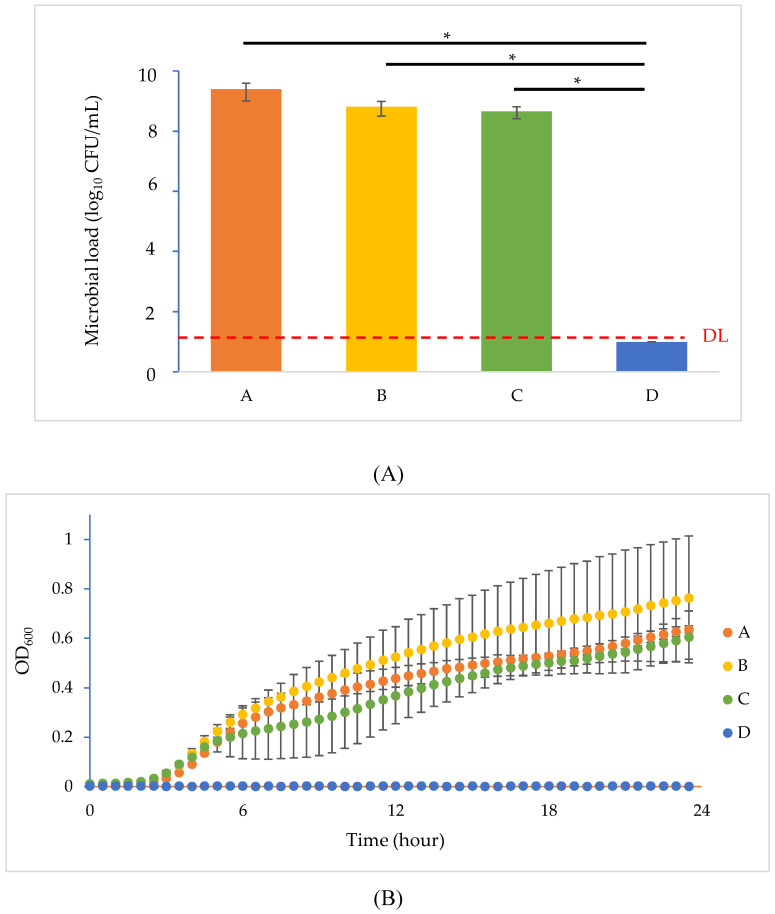
Phage resistance inhibition test. (**A**) Recovered microbial loads in four designed settings (A, B, C and D) after overnight incubation. Asterisks indicate statistical significance using ANOVA (*p* < 0.05). Detection limit (DL) is indicated with red dash line. (**B**) Bacterial growth curves in the settings of phage inhibition test over time. Each trial was performed in triplicate. (*) indicates significant differences between different settings (*p* < 0.05).

Given that all BIMs showed hypersensitivity to SDS [21], we evaluated the synergic effect of phage T4 and the sublethal amount of SDS (10 mg/mL) in suppressing the emergence of resistance. As shown in Figure 5A, after overnight incubation, settings containing either phage (setting B) or SDS (setting C) alone showed a similar recovered bacterial load as the control (about 9 log_10_ CFU/mL), while the bacterial rate in setting D was significantly lower than the other settings (*p* < 0.05), showing a 5-log reduction compared to the initial inoculum. This finding was supported by the result that the visual appearances of culture tubes A, B and C appeared to be cloudy, but tube D remained clear after incubation (Figure S1). Based on the recovery rates and visual confirmation, it was obvious that the inoculated bacteria populated readily in settings A, B and C but were completely inhibited in setting D. The growth curve study (Figure 5B) further supported this argument by characterizing bacterial growth over time. Bacterial proliferation in settings A, B and C had a lag phase of 3 h and showed similar OD_600_ values throughout the trial (*p* > 0.05). The similar bacterial growths in settings A and B suggested that using phage T4 alone had a limited effect on suppressing bacterial growth, possibly owing to the emergence of BIMs in setting B. However, in the presence of the T4–SDS combination, the concentration of bacterial cells in setting D was not significantly different from the bacteria-free negative control (*p* > 0.05). These findings demonstrate that the combination of phage and surfactant exerted a synergic antimicrobial effect not only suppressing the emergence of phage resistance but also eliminating bacterial growth which neither phage nor SDS alone could achieve.

## 3. Discussion

The rise of phage-resistant mutants is one of the major obstacles limiting the efficacy of phage application. It is believed that using phages simultaneously with antibiotics can suppress the emergence of phage resistance and thus improve the antimicrobial effect of phages [21,26]. However, little is known about the physiological changes of mutants after acquiring phage resistance and if other food-grade substrates can be incorporated to enhance the efficacy of phage-based food safety interventions. In this study, while T4 attached to the WT *E. coli* B rapidly as previously described [27,28], six BIMs resisted T4 adsorption and therefore prevented subsequent formation of a lysis zone. The identified mutation in glycosyltransferase WaaG and the comparative LPS glycosyl composition analysis collectively suggested that the receptor glucose in BIMs’ LPS was truncated, consequently conferring resistance by preventing phage T4 from adsorbing to the bacterial surface. However, this truncation in the LPS increased the OM permeabilities to different compounds, especially to the food-grade surfactant SDS. By exploiting the hypersensitivity to SDS, the design of phage–SDS combination not only successfully eliminated the inoculated bacteria but also suppressed the emergence of phage resistance.

*Escherichia coli* B is a common laboratory strain which has been used for studying bacteriophages since the 1920s [29]. Its interaction with phage T4 has been characterized by extensive studies [22,23,27,30,31]. It is a common perception that T4 uses both the LPS and outer membrane protein C (ompC) as the receptor while infecting *E. coli* [32]. However, in the case of *E. coli* B, phage T4 only absorbs to the receptor site (glucose) in the LPS because ompC expression is absent in this particular strain [23,33]. Therefore, this classic model system allows us to specifically study the impact of phage-induced LPS truncation on the OM structure by excluding the influence of general porin ompC.

It is known that phage T4 tail fiber protein gp 37 interacts with the glucose terminus in the outer core area of the LPS and then anchors the phage particle onto the *E. coli* B surface [22]. Mutations in the chromosomal operon waa (formerly known as rfa), which contains a cluster of structural genes that are responsible for the biosynthesis of LPSs, have been associated with the loss of phage receptors due to LPS truncation [34,35,36]. In this study, we identified six-amino acid deletion in glucosyltransferase WaaG which is responsible for transferring the first phage-binding glucose to the LPS. Recently, Liebau et al. constructed the 3D structure of protein WaaG isolated from *E. coli* K-12 W3110 (PDB accession number 2IW1) and characterized its membrane interaction (Figure S2) [37]. Most importantly, they identified a membrane-interacting region in the N-terminal domain of WaaG (MIR-waaG) which anchors this enzyme to the cytosolic side of the *E. coli* inner membrane [37]. This putative MIR-waaG was proposed as a 30-amino acid region (YAEKVAQEKGFLYRLTSRYRHYAAFERATF) situated at residues 103-132 in the WaaG sequence. Comparative gene alignment (Figure 3) showed that the K-12 WaaG sequence showed a high degree of protein similarity to that of the WT *E. coli* B (identities = 90%, coverage = 100%, and E-value = 0) used in this study; an almost identical sequence of MIR-waaG was found in *E. coli* B at the same position of residues 103-132. Thus, it is conceivable that the *E. coli* B WaaG possesses a highly homologous and functional membrane-interacting region as the K-12 WaaG. Interestingly, the putative membrane-interacting region in *E. coli* B WaaG overlapped two hydrophobic amino acids (tyrosine [Y] and alanine [A]) with the deletion region (ADVCYA) in BIMs’ waaG sequences (Figure 3). Since hydrophobic interactions in the N-terminal domain are considered as the key contributors to glycosyltransferase membrane anchoring [37,38], the loss of the first two hydrophobic amino acid residues in BIM’s MIR-waaG might obstruct the enzyme anchoring to the inner membrane, and thus inactivating the reaction of glucose transfer and resulting in the loss of phage T4 receptor.

In the LPS sugar composition analysis, although a trace amount of glucose was still detected, possibly due to the residual biofilm which also accounts for the presence of mannose [39,40], the substantial difference in glucose content between the LPS from WT *E. coli* B and ZZa3 validated the glucose truncation of the LPS inner core, conferring a phenotype similar to the *waaG* mutant derived from *E. coli* F470 [34]. More importantly, Yethon et al. pointed out that the *waaG* mutation could not only trim the LPS but also destabilize the entire OM structure by discouraging the phosphorylation of heptose residues in the inner core [34]. As an effective permeability barrier, the negatively charged phosphate substituents in the LPS are essential for binding to divalent cations which laterally cross-link the neighboring LPS molecules. The fact that a total of 80% reduction in heptose phosphorylation was observed in the *waaG* mutant [34] could substantially disrupt the cross-linkage among neighboring LPS molecules and permeabilize the OM [41]. This loss of lateral strength among LPS molecules likely permeabilized the membrane of BIMs and led to the decreases in MIC values of BIMs to most tested compounds (Table 2) since ResFinder discovered no difference in the antibiotic resistance gene profiles between the parental strain and six mutants.

To maximize the chance of survival, bacteria alter their OM structure on environmental cues. Upon phage T4 infection, *E. coli* B mutants survived by removing the phage receptor site glucose on the LPS chain. As a cost of this modulation, the OM of these BIMs became highly permeable to the surfactant SDS. In the phage resistance inhibition test, high fluctuation of OD_600_ values was observed in setting B, possibly due to the varied bacteria–phage interactions in each independent trial. Similar to the antibiotic–phage synergy (PAS) reported previously [21], our results collectively showed that the T4–SDS combination significantly reduced bacterial growth and suppressed phage resistance. This is also consistent with the observations by Scanlan et al. [42]. However, the same study also argued that the combination of phage and surfactant did not necessarily achieve a synergic antibacterial effect [42]. They tested the combination of SDS with three phages (T4, λcl6 and T7) from different families of Caudovirales and showed that except for phage T4, the presence of a small amount of SDS (0.3 mg/mL) would actually negatively affect the survivals of *Siphoviridae* phage λcl6 and *Podoviridae* phage T7 in liquid media, and thus buffer the antibacterial effects. Similar results were observed in the combination of bile salt with phages [42,43]. This evidence of varied synergic effects suggests that caution must be taken in future studies while selecting the specific combination of phage and surfactant, since the success of the synergic effect depends on not only the bacterial target but also the potential interactions between selected phages and surfactants.

Not only a cleaning and hygienic detergent, SDS is also generally a food additive that can be safely used in egg white products, marshmallows and fruit juice [44,45]. Although our phage resistance inhibition design was able to achieve a 5-log reduction and suppress phage resistance, other aspects need to be considered. For example, the maximum limit of SDS allowed in food (solid egg white) is ten times less than the dose used in this study [45]. Moreover, further experiments are required to test this method on different food matrices in which the influences of food properties and environmental factors on the synergic effect remain elusive. Most importantly, the antibacterial impact on pathogenic *E. coli* strains might not be as straightforward as in *E. coli* B. Preliminary studies for *E. coli* O157:H7 BIMs found various mutations in genes related to receptors OmpC and LPS, suggesting inconsistent membrane permeabilities which would render the antimicrobial outcome of the combination of phages and a secondary substrate ineffective [46].

Having been exposed to phage T4, *E. coli* BIMs harboring a mutation in *waaG* acquired phage resistance by detaching glucose residues from their LPS. However, this truncation of the LPS came with a costly change in OM permeability including the hypersensitivity to SDS. The T4–SDS combination designed in this study was shown to be successful in eliminating phage resistance and bacterial growth simultaneously. These findings thereby evidence that the combination of a phage and sublethal amount of a food-grade surfactant could be a potential novel phage-based food safety intervention.

## 4. Materials and Methods

### 4.1. Bacterial Strain and Growth Condition

*Escherichia coli* B ATCC 11303 was cultured at 37 °C for 18 h in Luria-Bertani broth (LB; Sigma-Aldrich, St-Louis, Missouri, MO, USA) in an orbital shaker at a speed of 225 rpm.

### 4.2. Bacteriophage T4 Preparation

Bacteriophage T4 was purchased from American Type Culture Collection (Manassas, VA, USA). High-titre phage stocks were prepared as previously described [47]. Briefly, approximately 10^4^ PFU T4 particles were added to 4 mL of molten agar (LB with 0.8% agar) containing 100 µL of *E. coli* B overnight culture and overlaid onto a pre-warmed LB agar plate (LB with 1.5% agar). After incubation at 37 °C for 18 h, the top agar containing phages was scraped off and submerged in 20 mL lambda buffer to allow phage elution, followed by centrifugation at 6000× *g* for 10 min at 4 °C. This crude phage lysate was further purified with DNase (Roche Diagnostics, Mannheim, Germany), RNase (Roche Diagnostics, Germany) and Proteinase K (Roche Diagnostics, Germany), then concentrated by adding the precipitator polyethylene glycol (Fisher Scientific, Fair Lawn, NJ, USA).

### 4.3. BIMs Isolation and Confirmation

Bacteriophage-insensitive mutants were generated using the double agar overlay method by Kropinski, et al. [48]. Briefly, a uniform bacterial lawn was formed by overlaying 4 mL molten agar (LB with 0.8% agar; Sigma-Aldrich, St. Louis, MO, USA) inoculated with 100 µL of the wildtype *E. coli* B overnight culture onto an LB agar plate (Sigma-Aldrich, St. Louis, MO, USA). After the molten agar was solidified, 100 µL of each serial diluted phage T4 lysate (from 10^10^ to 10^0^ PFU/mL) was dispensed onto individual bacterial lawns and incubated at 37 °C for 18 h. Colonies that appeared within the phage clearing after incubation were considered as potential BIMs. Phage resistance of these candidates was further confirmed by a spot test as described by Clokie, et al. [49]. Ten microliters of high-titre phage T4 lysate (10^10^ PFU/mL) was spot-inoculated on the bacterial lawn populated with each putative BIM. The resistance to phage T4 can be finally confirmed by the lack of lysis at the phage-infected spots.

### 4.4. Phage Adsorption to Bacterial Cells Measurement

The adsorption curves of phage T4 to the wildtype and each BIM were determined as described by Kropinski [50]. Briefly, a mid-logarithms phase culture of the target bacterium was diluted into LB broth supplemented with 10 mM calcium chloride to give OD_600_ of 0.3, followed by addition of phage T4 at a multiplicity of infection (MOI) of 0.01. At several timepoints (1, 2, 4, 7 and 10 min), an aliquot was taken from the mixture and further diluted into a prechilled tube with the same media and three drops of chloroform. The titre of unadsorbed phages in each collected sample was then enumerated using the double agar overlay method, proliferating with the wildtype *E. coli* B overnight. A nonadsorbing control in the absence of bacteria was used to determine the initial phage titre which was then set to 100%. Each assay was performed in duplicate and repeated at least three times. Phage adsorption rate constants were calculated using the following Equation (1) [51]:

(1) $$k=\frac{2.3}{Bt}\log\frac{P_{0}}{P^{\prime }}$$

where *k* represents the adsorption rate constant (mL/min); B stands for the inoculated bacterial load; and *t* is the time (min) required for a phage titre change from initial *P*_0_ to final *P*.

### 4.5. DNA Extraction, Whole Genome Sequencing and Bioinformatic Analysis

Genomic DNA of the wildtype *E. coli* B and BIMs were extracted from LB broth cultures after 37 °C overnight incubation using the E-Z 96 Tissue DNA Kit (Omega Bio-tek, Norcross GA, USA) following the manufacturer’s instructions. Approximately 500 ng of genomic DNA was extracted and mechanically fragmented for 40 s by Covaris M220 (Covaris, Woburn MA, USA) using the default settings. Libraries were synthesized using the NEBNext Ultra II DNA library prep kit for Illumina (New England Biolabs, Ipswich MA, USA) according to the manufacturer’s instructions and were sequenced using the EcoGenomics Analysis Platform on an Illumina^®^ MiSeq sequencer with TruSeq 300 bps paired-end libraries and 30 × coverage giving approximately 75 contigs per genome.

### 4.6. Bioinformatic Analysis

The raw reads of sequenced strains were de novo assembled using the A5-miseq pipeline [52]. The sequence of the wildtype *E. coli* B was used as a reference for alignment and comparison to the BIMs. Retrieved genomes were then annotated by the Rapid Annotation using Subsystem Technology (RAST) service [53]. Single nucleotide polymorphisms detection was performed using the Geneious software. Genome comparison was performed using the MAUVE software v2.3.1. Gene sequences were aligned using the online platform Clustal Omega Multiple Sequence Alignment and visualized by Jalview version 2.11.0 [54,55]. Lastly, ResFinder was used to compare the acquired antibiotic resistance profiles between the wildtype and BIMs [56].

### 4.7. Isolation of LPS and Glycosyl Composition Analysis

LPSs were prepared using the hot phenol-water extraction procedure [57]. Essentially, bacterial cells were collected via centrifugation and resuspended into molecular-grade water. The same volume of preheated 90% phenol was added to extract the LPSs at 70 °C for 20 min. The mixture was then cooled down in ice and centrifuged at 5000× *g* at 4 °C to promote phase separation. The upper (aqueous) phase of the mixture was collected. Extraction was repeated by adding the same volume of water into the lower (organic) phase of the mixture three times. Next, crude LPSs in the water phase were dialyzed (6000 MWCO) at 4 °C against water until no detectable phenol remained, followed by treatments with RNase, DNase and proteinase K. The purified LPSs were dialyzed again and finally ultra-centrifuged at 100,000× *g* at 4 °C for 18 h. Both LPS pellets and supernatants were collected individually for the following analysis.

The sugar components of the LPS from the wildtype and one of the BIMs, ZZa3, were determined. Extracted LPSs were converted to trimethylsilyl (TMS) methylglycosides through methanolysis where 1M HCl in methanol was added to the sample in the presence of an internal standard inositol and held at 80 °C for 18 h. The TMS derivatives were analyzed by GC-MS as previously described [58,59] on a HP5890 gas chromatograph (Hewlett-Packard, Wilmington, Delaware, USA) equipped with a mass selective detector 5970 (Hewlett-Packard, USA) using an EC-1 fused silica capillary column (30 m × 0.25 mm I.D.). Temperature cycle started at 80 °C for 2 min, then ramped to 160 °C at a rate of 20 °C/min, and to 200 °C at 2 °C/min, followed by an increase to 250 °C at 10 °C/min with an 11-min hold.

### 4.8. Minimum Inhibitory Concentration Assays

Minimum inhibitory concentration assays were conducted to compare the susceptibilities of the wildtype strain and six BIMs to various compounds, i.e., kanamycin sulfate (Sigma-Aldrich, USA), ampicillin (Sigma-Aldrich, St. Louis, MO, USA), ethylenediaminetetraacetic acid (EDTA, ThermoFisher, Rockford, IL, USA), novobiocin (Sigma-Aldrich, St. Louis, MO, USA), polymyxin B sulfate (ThermoFisher, Waltham, MA, USA), sodium deoxycholate sulfate (ThermoFisher, Waltham, MA, USA) and SDS (ThermoFisher, Waltham, MA, USA), as previously described [34].

Briefly, minimum inhibitory concentration assays were carried out in culture tubes where each contained 5 mL of LB broth. Two-fold serial dilutions of kanamycin sulfate (from 64 to 1 μg/mL), ampicillin (from 64 to 1 μg/mL), novobiocin (from 200 to 1 μg/mL), polymyxin B sulfate (from 200 to 1 μg/mL), EDTA (from 200 to 1 mg/mL), sodium cholate sulfate (from 128 to 1 mg/mL) and SDS (from 200 to 1 mg/mL) were made in these tubes. Each series of tubes was inoculated with 100 μL of overnight *E. coli* B cultures and incubated with orbital shaking at 225 rpm at 37 °C for 18 h. A positive score was recorded if the culture was visibly turbid. The MIC of each isolate to a certain compound was determined based on the tube with the highest concentration and visibly clear appearance. Each trial was performed in duplicate and repeated on three individual experiments.

### 4.9. Phage Resistance Inhibition Test

Since BIMs conferred hypersensitivity to the anion detergent SDS, a phage resistance inhibition test consisting of four settings (A, B, C and D) was conducted as follows (Table 3). This experiment was performed simultaneously in culture tubes and a 96-well microplate. Each setting was inoculated with an equal amount of *E. coli* B. While setting A was used as a negative control, setting B and C described the solo impact of phage T4 and low level of SDS on bacterial growth, respectively. Lastly, in setting D, both phage T4 and a sublethal amount of SDS were used to assess the synergic effect of this combination on suppressing the emergence of phage resistance. After overnight incubation with shaking at 225 rpm at 37 °C, 100 μL of culture from each setting was enumerated using spread plating to determine the bacterial loads (detection limit = 10 CFU/mL) and the visual appearance of these four tubes was also captured. In the parallel study, the microbial growth in the designed settings was recorded at OD_600_ using a Synergy HTX Multi-Mode Reader (Winooski, VT, USA). Each trial was performed in duplicate and repeated on three individual experiments.

### 4.10. Statistical Analysis

Single factor ANOVA was used to detect differences in bacterial growth in the four settings of the phage resistance inhibition test. Data were analyzed using Microsoft Excel and a difference was considered significant at *p* < 0.05.

## Figures and Tables

**Table 1:** Glycosyl composition analysis of LPSs isolated from *E. coli* B WT and ZZa3 mutant.

Strain	LPS Glycosyl Composition [Mole %]					
	Rib	Man	Glc	Hep	GlcN	Kdo *
WT *E. coli* B	12.2	4.3	17.8	62.2	3.5	+
ZZa3	12.8	2.1	1	78.5	5.7	+

* Due to unavailability of Kdo standard, the relative mole % of Kdo was not included in the calculation.

**Table 2:** Minimum inhibitory concentration assays for the WT *E. coli* B and BIMs.

Substrates	WT	ZZa0	ZZa1	ZZa2	ZZa3	ZZa4	ZZa5
Ampicillin (µg/mL)	4	2	2	2	2	1	1
Kanamycin (µg/mL)	32	16	16	16	16	16	16
Novobiocin (µg/mL)	>200	>200	>200	>200	>200	>200	>200
Polymyxin B (µg/mL)	64	32	32	32	32	32	32
EDTA (mg/mL)	50	25	25	25	25	25	25
Sodium Cholate (mg/mL)	>128	128	128	128	128	128	128
SDS (mg/mL)	>200	3	1.5	3	3	3	1.5

**Table 3:** Design of the phage resistance inhibition test.

	Setting A	Setting B	Setting C	Setting D
*E. coli* B (10^6^ CFU)	+	+	+	+
Phage T4 (10^7^ PFU)	−	+	−	+
SDS (10 mg/mL)	−	−	+	+

## Supplementary Materials

The following are available online at https://www.mdpi.com/2079-6382/9/9/552/s1, Figure S1: Visual appearances of the four settings in the phage resistance inhibition tests, Figure S2: Model of WaaG from *Escherichia coli* K-12 anchoring to simulated membrane (grey). Crystal structure of glycosyltransferase waaG (PDB accession number 2IW1) is in blue and MIR-waaG is in orange.
