# Supplementary material for: Bacteriophage-Induced Lipopolysaccharide Mutations in Escherichia coli Lead to Hypersensitivity to Food Grade Surfactant Sodium Dodecyl Sulfate
Source: Antibiotics (Basel). 2020 Aug 28;9(9):552. doi: 10.3390/antibiotics9090552 (PMC7558818; doi:10.3390/antibiotics9090552)
Supplement: Supplementary file 1 [file antibiotics-09-00552-s001.pdf]

Supplemental Materials

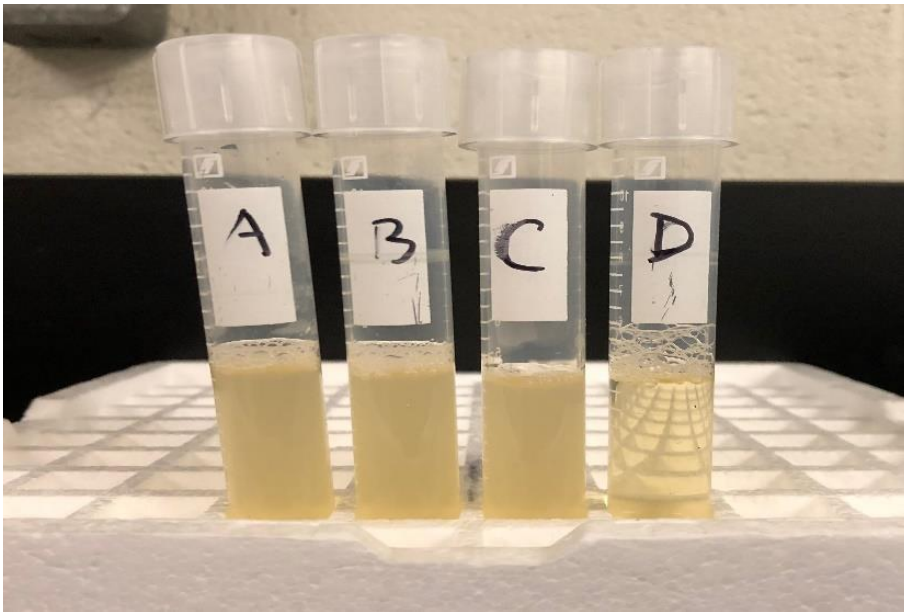

Figure S1. Visual appearances of four settings in phage resistance inhibition tests.

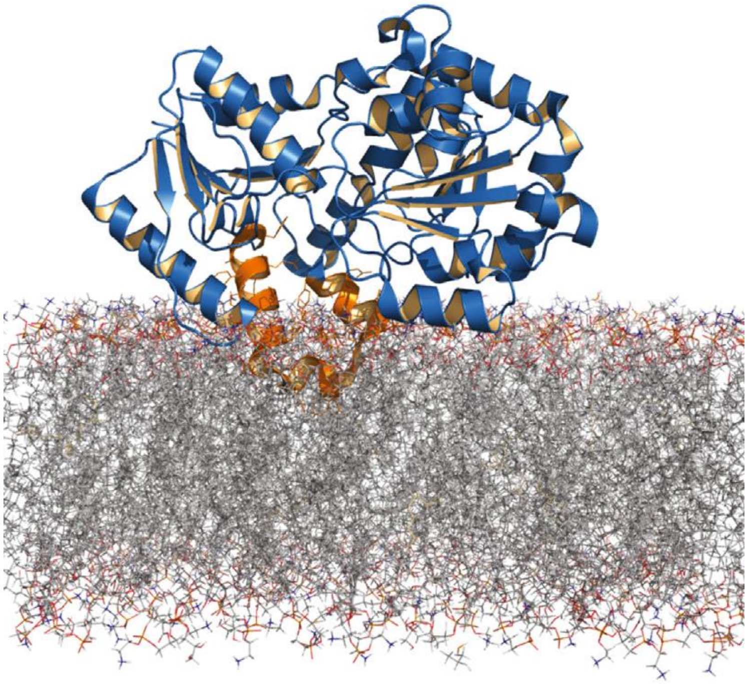

Figure S2. Model of WaaG from *Escherichia coli* K-12 anchoring to simulated membrane (grey). Crystal structure of glycosyltransferase waaG (PDB accession number 2IW1) is in blue and MIR-waaG is in orange. This figure was adapted from Liebau et al.
